# Evaluation of the integration of telehealth into the same‐day antiretroviral therapy initiation service in Bangkok, Thailand in response to COVID‐19: a mixed‐method analysis of real‐world data

**DOI:** 10.1002/jia2.25816

**Published:** 2021-10-28

**Authors:** Sorawit Amatavete, Sita Lujintanon, Nipat Teeratakulpisarn, Supanat Thitipatarakorn, Pich Seekaew, Chonticha Hanaree, Jirayuth Sripanjakun, Chotika Prabjuntuek, Lertkwan Suwannarat, Thana Phattanathawornkool, Nuttawoot Photisan, Sujittra Suriwong, Matthew Avery, Stephen Mills, Praphan Phanuphak, Nittaya Phanuphak, Reshmie A. Ramautarsing

**Affiliations:** ^1^ Institute of HIV Research and Innovation Bangkok Thailand; ^2^ Department of Epidemiology Columbia University Mailman School of Public Health New York USA; ^3^ FHI 360 and LINKAGES Bangkok Thailand

**Keywords:** HIV, same‐day antiretroviral therapy, differentiated care, telehealth, linkage to care COVID‐19, Asia

## Abstract

**Introduction:**

Same‐day antiretroviral therapy (SDART) initiation has been implemented at the Thai Red Cross Anonymous Clinic (TRCAC) in Bangkok, Thailand, since 2017. HIV‐positive, antiretroviral therapy (ART)‐naïve clients who are willing and clinically eligible start ART on the day of HIV diagnosis. In response to the first wave of the coronavirus disease 2019 (COVID‐19) outbreak in March 2020, telehealth follow‐up was established to comply with COVID‐19 preventive measures and allow service continuation. Here, we evaluate its implementation.

**Methods:**

Pre‐COVID‐19 (until February 2020) clients who initiated SDART received a 2‐week ART supply and returned to the clinic for evaluation before being referred to long‐term ART maintenance facilities. If no adverse events (AEs) occurred, another 8‐week ART supply was provided while referral was arranged. During the first wave of COVID‐19 (March–May 2020), clients received a 4‐week ART supply and the option of conducting follow‐up consultation and physical examination via video call. Clients with severe AEs were required to return to TRCAC; those without received another 6‐week ART supply by courier to bridge transition to long‐term facilities. This adaptation continued post‐first wave (May–August 2020). Routine service data were analysed using data from March to August 2019 for the pre‐COVID‐19 period. Interviews and thematic analysis were conducted to understand experiences of clients and providers, and gain feedback for service improvement.

**Results:**

Of 922, 183 and 321 eligible clients from the three periods, SDART reach [89.9%, 96.2% and 92.2% (*p* = 0.018)] and ART initiation rates [88.1%, 90.9% and 94.9% (*p*<0.001)] were high. ART uptake, time to ART initiation and rates of follow‐up completion improved over time. After the integration, 35.3% received the telehealth follow‐up. The rates of successful referral to a long‐term facility (91.8% vs. 95.3%, *p* = 0.535) and retention in care at months 3 (97.5% vs. 98.0%, *p* = 0.963) and 6 (94.1% vs. 98.4%, *p* = 0.148) were comparable for those receiving in‐person and telehealth follow‐up. Six clients and nine providers were interviewed; six themes on service experience and feedback were identified.

**Conclusions:**

Telehealth follow‐up with ART delivery for SDART clients is a feasible option to differentiate ART initiation services at TRCAC, which led to its incorporation into routine service.

## 1 INTRODUCTION

Same‐day antiretroviral therapy (SDART) initiation in which HIV treatment is started on the same day as HIV diagnosis is a safe and promising intervention to accelerate linkage to care. SDART is endorsed by the World Health Organization as a strategy in ending the HIV epidemic [1]. However, since available healthcare resources have been allocated to testing, treatment and mitigation of the coronavirus disease 2019 (COVID‐19) pandemic, the decreased rates of HIV testing [2, 3, 4], pre‐exposure prophylaxis [5, 6] and post‐exposure prophylaxis prescription [7, 8, 9], as well as antiretroviral therapy (ART) dispensation [4] were reported. With the increased perceived risk and fear of acquiring COVID‐19, many clients viewed these HIV services as non‐essential [10, 11], which resulted in clients not accessing the services.

In Thailand, the first wave of the COVID‐19 outbreak occurred during January–July 2020 [12] with a total of 3298 confirmed cases and 58 deaths [13]. The number of new infections escalated in March 2020 [14], which promptly led the Thai Government to issue a National Emergency Decree [15] and a nationwide curfew [16]. The implementation of these policies contributed greatly to the fall of local transmission in April 2020 and a drop to near zero cases in mid‐May 2020 [17]. During this time in Thailand, new governmental recommendations were launched to support the adaptation of HIV and related services to allow their continuation [18, 19, 20]. However, no recommendation on ART initiation service was put forth. Timely ART initiation was already challenging pre‐COVID‐19 epidemic due to difficulty in obtaining baseline laboratory results and a requirement for multiple pre‐ART counselling sessions to promote long‐term adherence. Despite HIV care being free in Thailand, people living with HIV (PLHIV) can only access free services at the specific healthcare facility where they are registered through their national health insurance and, in some cases, may require to change their facility coverage to another more convenient facility. These complicated requirements might contribute to loss to follow up [21, 22, 23, 24], adverse clinical events [25] and onward HIV transmission prior to ART initiation [25, 26] that have been reported worldwide. The added barriers of social distancing, provincial border lockdown and avoiding of non‐essential hospital visits during the COVID‐19 pandemic were anticipated to aggravate linkage to care [27].

The Institute of HIV Research and Innovation (IHRI) has piloted the SDART initiation service at the Thai Red Cross Anonymous Clinic (TRCAC) since July 2017. It was the first SDART initiation hub in the country where ART‐naïve, HIV‐diagnosed people who were willing and clinically eligible could start ART on the same day as HIV diagnosis free of charge, regardless of their insurance coverage. This service was provided by a multidisciplinary team of non‐specialist physicians, nurses, pharmacists, counsellors and peer navigators. The navigators, including but not limited to men who have sex with men (MSM), transgender women (TGW) and PLHIV, played an essential role in assisting PLHIV in retaining in care and overcoming the health system barriers. In early March 2020, the SDART provider team foresaw the aforementioned barriers of the COVID‐19 pandemic to SDART initiation and planned service adaptations to reduce the risk of COVID‐19 spread while optimizing linkage to care. This became the first differentiated ART initiation model available under the changed reality of health service delivery in the COVID‐19 period.

This study evaluates the integration of telehealth into the SDART initiation service at TRCAC in Bangkok, Thailand, by describing service outcomes in the pre‐, during and post‐first waves of the COVID‐19 epidemic and comparing the clinical outcomes of clients who received in‐person and telehealth follow‐up.

## 2 METHODS

### 2.1 Study design and participants

This is a sub‐study of an observational cohort study of all clients who tested HIV positive and underwent the routine SDART initiation service at TRCAC, which is a standalone HIV/sexually transmitted infection testing centre and an SDART initiation hub located in the centre of Bangkok, Thailand. This analysis evaluated the outcomes of the SDART initiation service from three periods: pre‐ (1 March 2019–31 August 2019), during (1 March 2020–15 May 2020) and post‐first waves of the COVID‐19 epidemic (16 May 2020–31 August 2020). All clients who tested HIV positive at TRCAC were screened for SDART eligibility: being ART‐naïve, not participating in another study and ability to attend follow‐up visit (pre‐COVID‐19 epidemic only). Eligible clients were included in this analysis.

### 2.2 SDART initiation procedure pre‐COVID‐19 epidemic

The SDART initiation procedure started after the client received the first positive HIV result (Architect HIV Ag/Ab Combo, Abbott, Germany, or Elecsys HIV combi PT, Roche Diagnostics GmbH, Germany) at TRCAC. The clients received post‐test counselling and were assessed for eligibility and willingness to start SDART by the counsellor. Those who consented received phlebotomy for HIV confirmatory [Rapid Test for Anti‐HIV (Colloidal Gold Device), Beijing Wantai Biological Pharmacy Enterprise Co., Ltd., China, and Serodia HIV‐1/2, Fujirebio Inc., Japan] and baseline pre‐ART laboratory tests (CD4 cell count, complete blood count, alanine aminotransferase, creatinine/creatinine clearance, urine analysis, Treponema pallidum hemagglutination, rapid plasma reagin, hepatitis B surface antigen, hepatitis C antibody and cryptococcal antigen for those with CD4 count < 100 cells/mm^3^). The clients travelled to receive a chest X‐ray at a nearby hospital. Afterwards, the clients met with a peer navigator to receive HIV diagnosis confirmation, screening for psychosocial readiness using Patient Health Questionnaire‐9 and pre‐ART initiation counselling, including adherence counselling. The date that this process takes place is referred to as the care engagement date, which due to logistics might not be on the same date as HIV diagnosis. A nurse and a physician then collected medical history and performed a physical examination to rule out tuberculosis (TB), cryptococcal meningitis and other serious illnesses that might interfere with ART initiation. GeneXpert MTB/RIF assay was performed for clients who were suspected of TB. If serious opportunistic infections (OIs) or illnesses were suspected, clients were referred to their registered healthcare facility for OI investigation, treatment and/or ART initiation. Clients who were clinically eligible were prescribed ART (tenofovir disoproxil fumarate 300 mg, emtricitabine 200 mg and efavirenz 600 mg once daily) as per national guidelines [28].

After SDART initiation, clients were scheduled for a follow‐up visit after 2 weeks, during which they received baseline laboratory results, physical examination and ART side effect assessment and/or management. Adverse events (AEs) included grades 1–3 [29]. If needed, ART regimen was modified. Otherwise, ART refill was provided, and the referral process was initiated in which the navigator assisted in the change in facility coverage process and accompanied the clients to their long‐term ART maintenance facility upon request. The SDART process, from ART initiation to referral, lasted approximately 2.5 months for each client. After referral, the navigator continued to follow up the clients remotely by calling, messaging and/or checking their ART status in the online national HIV database, NAPPLUS, to confirm successful referral to the ART maintenance facility. The navigator will also follow up with clients to assess their retention for up to 2 years after ART initiation.

Those diagnosed with HIV but were ineligible for SDART or were eligible but not willing to start SDART received confirmatory HIV tests and were referred to their preferred hospital with the assistance of the navigator.

### 2.3 Adaptation of SDART initiation service in response to COVID‐19

**Figure 1:**
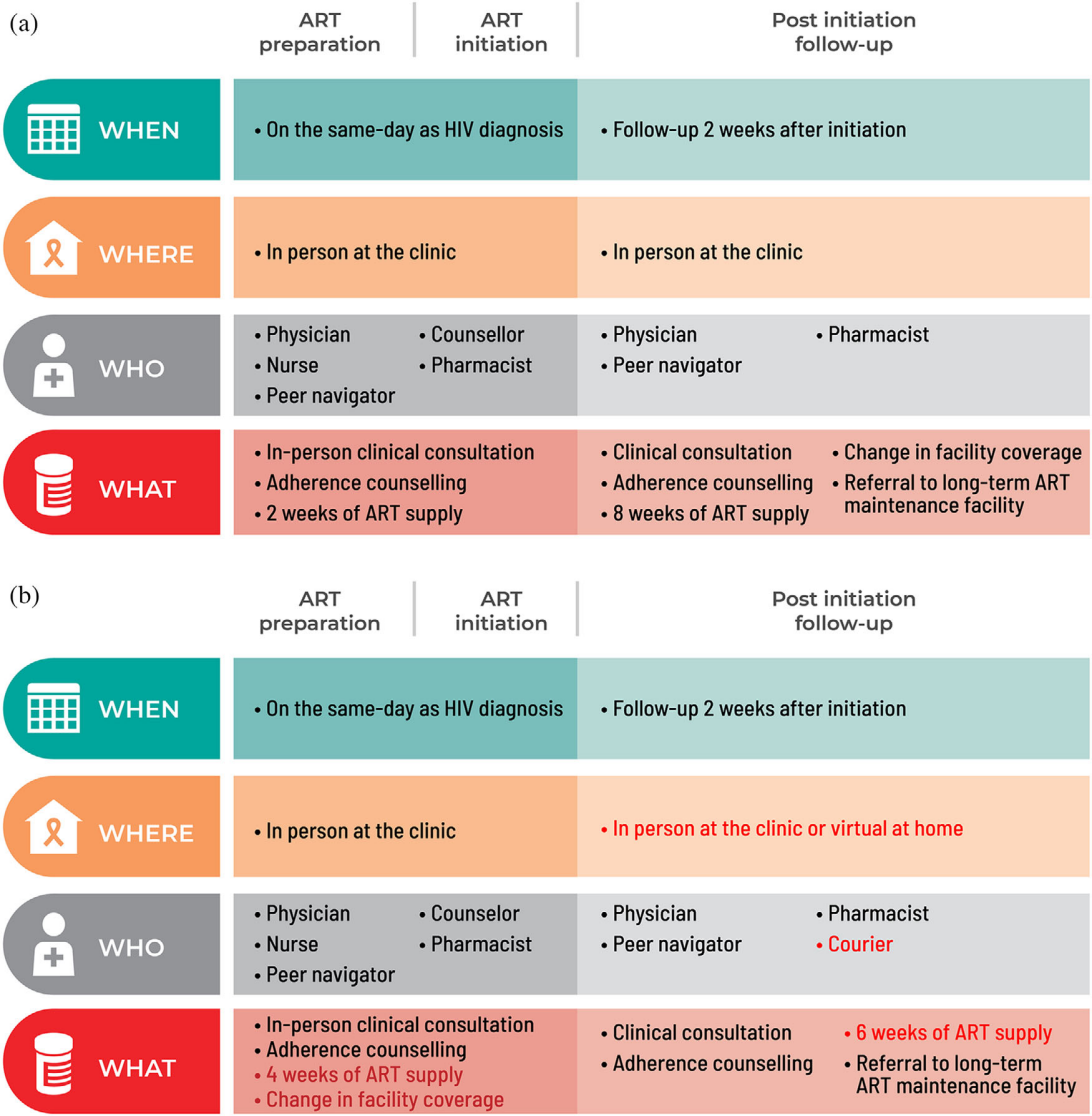
Differentiated same‐day antiretroviral therapy (SDART) initiation service before and in response to coronavirus disease 2019 (COVID‐19). The components of SDART initiation service models before (a) and in response to (b) COVID‐19 are presented according to the differentiated service delivery framework with the red text indicating where the adaptation occurred. The service is divided into three parts: ART preparation, ART initiation and post initiation follow‐up, with each part describing the timing, location, provider and frequency of services delivered. Abbreviation: ART, antiretroviral therapy.

**Figure 2:**
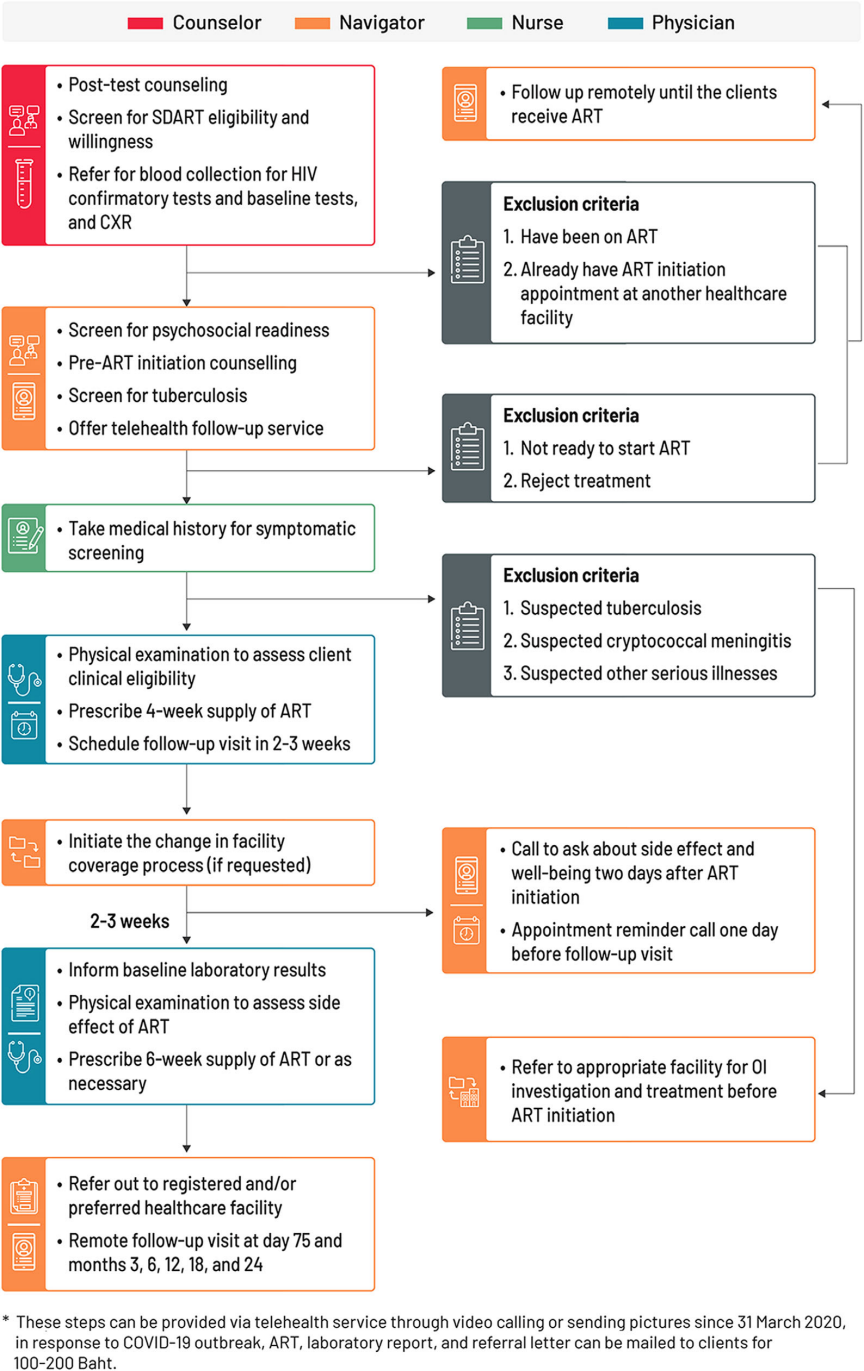
Same‐day antiretroviral therapy (SDART) initiation service flow during and post‐first waves of the coronavirus disease 2019 (COVID‐19) epidemic. The SDART initiation service flow outlines the tasks conducted by the four main teams of SDART providers, which are counsellors, peer navigators, nurses and non‐specialist physicians, from the ART initiation visit to the follow‐up visit and the remote follow‐up processes. Abbreviations: ART, antiretroviral therapy; CXR, chest X‐ray; NAPPLUS, National AIDS Program Plus; OI, opportunistic infection; SDART, same‐day antiretroviral therapy.

The SDART initiation service models before and in response to COVID‐19 are presented according to the differentiated service delivery (DSD) framework [30] in Figure 1. The ability to attend a follow‐up visit at TRCAC was dropped from the eligibility criteria as the telehealth follow‐up option was added to allow follow‐up via video call using the LINE application. This application has been very popular in Thailand for instant communication with free audio and video calls. Those who lacked the skills in using the application or had limited access to high‐speed internet for video calls were allowed to use audio‐only calls and send photographs of additional laboratory test reports or their visible symptoms via LINE chat. ART refill was done via mail. The clients paid a delivery fee of 100 Thai baht (approximately US$3). The refill duration at the initiation visit was adjusted from 2 to 4 weeks to ensure adequate ART supply until the follow‐up visit. Insurance transfer was offered at the initiation visit instead of at follow‐up. The adapted SDART initiation service flow is shown in Figure 2.

### 2.4 Statistical analysis

Data were stratified into pre‐, during and post‐first waves of the COVID‐19 epidemic. Outcome measures included demographic characteristics, baseline CD4 cell count, SDART eligibility rate, SDART reach rate [31], ART initiation rate, duration to initiate ART, follow‐up visit completion rate, AE rate, duration to change facility coverage, referral completion rate and retention rates at months 3 and 6. Descriptive analysis summarized the client characteristics, service outcomes and clinical outcomes using proportions, mean, standard deviation, median and interquartile range. Pearson's chi‐square and Fisher's exact tests were used to determine the relationship between categorical variables. One‐way ANOVA was used to compare means between the three periods. Kruskal–Wallis equality‐of‐populations rank test was applied to test equality of median distribution across groups. An independent samples *t*‐test was used to compare means between in‐person and telehealth follow‐up groups. Univariate and multivariate logistic regression analyses were conducted to explore the associated factors with receiving telehealth follow‐up. A *p*‐value of <0.05 was considered as statistically significant.

Statistical analysis was conducted with Stata version 15.0 (StataCorp, College Station, TX, USA).

### 2.5 Qualitative assessment

In October 2020, after the telehealth follow‐up was continued as part of routine service delivery, a small subset of clients who completed telehealth follow‐up and SDART providers were interviewed to assess their experiences and feedback for the purpose of service improvement. Interview participants were conveniently selected to represent the clients and each cadre of providers until the point of data saturation. The clients were interviewed by the navigators via LINE chat, which is a communication channel already used to communicate and form rapport between clients and navigators. The providers were interviewed in‐person by navigators and program officers. The interview questions can be found in Appendix S1. Interview transcripts were generated, and thematic analysis was conducted manually by three program officers following the framework analysis outlined by Braun and Clarke. Each officer reviewed the entirety of the transcripts, generated codes from the relevant data and developed potential themes independently. Afterwards, they convened to discuss and finalize the themes, sub‐themes and quotes to demonstrate each sub‐theme [32]. Selected quotes in Thai were translated verbatim into English.

### 2.6 Ethical consideration

This study was approved by the Institutional Review Board of the Faculty of Medicine, Chulalongkorn University (IRB158/56). The informed consent was waived as the routine service data were collected as secondary data with no personal identifiers. Interviewed participants provided verbal consent.

## 3 RESULTS

A total of 1728 clients were screened for SDART eligibility during the study periods: 1084 pre‐, 238 during and 406 post‐first waves of the COVID‐19 epidemic. Of these, 922 (85.1%), 183 (76.9%) and 321 (79.1%) were eligible for SDART, respectively. Their characteristics are shown in Table 1.

**Table 1:** Characteristics of same‐day antiretroviral therapy (SDART) eligible clients in the pre‐, during and post‐first waves of the coronavirus disease 2019 (COVID‐19) epidemic

	Pre‐first wave (*N* = 922)	During the first wave (*N* = 183)	Post‐first wave (*N* = 321)	*p*‐value
Age (years), Mean (SD)	31.1 (9.3)	31.3 (10.0)	30.6 (8.2)	0.587*
Age group				0.581**
<25 years old	282/922 (30.6%)	50/183 (27.3%)	91/320 (28.4%)	
≥25 years old	640/922 (69.4%)	133/183 (72.7%)	229/320 (71.6%)	
Assigned sex at birth				0.965**
Male	802/922 (87.0%)	160/183 (87.4%)	278/321 (86.6%)	
Female	120/922 (13.0%)	23/183 (12.6%)	43/321 (13.4%)	
Population				0.449**
Heterosexual	219/922 (23.8%)	46/183 (25.1%)	76/321 (23.7%)	
MSM	658/922 (71.4%)	122/183 (66.7%)	227/321 (70.7%)	
TGW	45/922 (4.8%)	15/183 (8.2%)	18/321 (5.6%)	
Insurance				0.168**
UCS	380/913 (41.6%)	74/180 (41.1%)	139/319 (43.6%)	
SSS	371/913 (40.6%)	78/180 (43.3%)	130/319 (40.8%)	
CSMBS	52/913 (5.7%)	17/180 (9.4%)	16/319 (5.0%)	
Other schemes	2/913 (0.2%)	0/180 (0%)	0/319 (0%)	
Pay out of pocket	107/913 (11.7%)	10/180 (5.6%)	32/319 (10.0%)	
No scheme	1/913 (0.1%)	1/180 (0.6%)	2/319 (0.6%)	
CD4 cell count group (cells/mm^3)^				0.241**
≤100	125/922 (13.6%)	25/183 (13.7%)	52/321 (16.2%)	
101–349	408/922 (44.3%)	76/183 (41.5%)	154/321 (48.0%)	
≥350	389/922 (42.1%)	82/183 (44.8%)	115/321 (35.8%)	

* One‐way ANOVA.

** Pearson's chi‐square test.

Abbreviations: COVID‐19, coronavirus disease 2019; CSMBS, Civil Servant Medical Benefit Scheme; MSM, men who have sex with men; SD, standard deviation; SSS, Social Security Scheme; TGW, transgender women; UCS, Universal Coverage Scheme.

The service outcomes of eligible clients pre‐, during and post‐first waves of the COVID‐19 epidemic are shown in Table 2.

**Table 2:** Service outcomes of same‐day antiretroviral therapy (SDART) eligible clients in the pre‐, during and post‐first waves of the coronavirus disease 2019 (COVID‐19) epidemic

	Pre‐first wave (*N* = 922)	During the first wave (*N* = 183)	Post‐first wave (*N* = 321)	*p*‐value
SDART reach	829/922 (89.9%)	176/183 (96.2%)	296/321 (92.2%)	0.018*
ART initiation	730/829 (88.1%)	160/176 (90.9%)	281/296 (94.9%)	<0.001*
Median (Q1, Q3) duration from HIV diagnosis to ART initiation (days)	1 (0, 4)	1 (0, 2)	0 (0, 2)	<0.001**
Median (Q1, Q3) duration from care engagement to ART initiation (days)	1 (0, 2)	0 (0, 1)	0 (0, 1)	0.001**
Follow‐up visit completion	706/730 (96.7%)	157/160 (98.1%)	277/281 (98.6%)	<0.001*
In‐person follow‐up	706/706 (100%)	102/157 (65.0%)	179/277 (64.6%)	
Telehealth follow‐up	N/A	55/157 (35.0%)	98/277 (35.4%)	
Median (Q1, Q3) duration from care engagement to successful change in facility coverage (days)	17 [14, 21]	14.5 (0, 17)	0 (0, 12)	<0.001**
Referral to long‐term ART maintenance facility among those with ≥ 2.5 months follow‐up time				0.451*
Successful	663/706 (93.9%)	147/157 (93.6%)	254/277 (91.7%)	
Not Successful	43/706 (6.1%)	10/157 (6.4%)	23/277 (8.3%)	
Retention at month 3 among those reached month 3				0.666***
In care	678/706 (96.0%)	154/157 (98.1%)	265/277 (95.7%)	
LTFU	17/706 (2.4%)	1/157 (0.6%)	8/277 (2.9%)	
Discontinued ART	11/706 (1.6%)	2/157 (1.3%)	4/277 (1.4%)	
Retention at month 6 among those reached month 6				0.014***
In care	690/706 (97.7%)	151/157 (96.2%)	165/173 (95.4%)	
LTFU	11/706 (1.6%)	2/157 (1.3%)	8/173 (4.6%)	
Discontinued ART	5/706 (0.7%)	4/157 (2.5%)	0/173 (0%)	

* Pearson's chi‐square test.

** Kruskal–Wallis equality‐of‐populations rank test.

*** Fisher's exact test.

Abbreviations: ART, antiretroviral therapy; COVID‐19, coronavirus disease 2019; LTFU, loss to follow up; Q1, the first quartile; Q3, the third quartile; SDART, same‐day antiretroviral therapy.

Table 3 shows the characteristics of 434 clients who received in‐person follow‐up (64.7%) and telehealth follow‐up (35.3%) between 1 March and 31 August 31 2020. Univariate logistic regression analysis found no statistically significant factors associated with receiving telehealth follow‐up. Therefore, multivariate analysis was not conducted.

**Table 3:** Characteristics of clients and factors associated with receiving telehealth follow‐up

				Univariate logistic regression model	
				
	In‐person follow‐up (*N* = 281)	Telehealth follow‐up (*N* = 153)	*p*‐value	Crude OR (95% CI)	*p*‐value
Age (years), Mean (SD)	31 (8.9)	30 (8.3)	0.679*	1 (0.97–1.02)	0.678
Age group			0.804**		
<25 years old	80/280 (28.6%)	42/153 (27.5%)		0.95 (0.61–1.47)	0.804
≥25 years old	200/280 (71.4%)	111/153 (72.5%)		1	–
Assigned sex at birth			0.140**		
Male	246/281 (87.5%)	126/153 (82.4%)		1	–
Female	35/281 (12.5%)	27/153 (17.6%)		1.51 (0.87–2.60)	0.142
Population			0.336**		
Heterosexual	64/281 (22.8%)	44/153 (28.8%)		1	–
MSM	199/281 (70.8%)	98/153 (64.1%)		0.72 (0.46–1.13)	0.149
TGW	18/281 (6.4%)	11/153 (7.2%)		0.89 (0.38–2.06)	0.784
Insurance			0.375***		
UCS	119/281 (42.3%)	65/153 (42.5%)		1.06 (0.68–1.63)	0.806
SSS	116/281 (41.3%)	60/153 (39.2%)		1	–
CSMBS	19/281 (6.8%)	11/153 (7.2%)		1.12 (0.50–2.50)	0.784
Pay out of pocket	25/281 (8.9%)	16/153 (10.5%)		1.24 (0.61–2.49)	0.551
No scheme	2/281 (0.7%)	1/153 (0.6%)		0.97 (0.09–10.88)	0.978
CD4 cell count group (cells/mm^3^)			0.349**		
≤100	31/281 (11.0%)	20/153 (13.1%)		1.03 (0.54–1.97)	0.918
101–349	149/281 (53.0%)	70/153 (45.8%)		0.75 (0.49–1.15)	0.190
≥350	101/281 (35.9%)	63/153 (41.2%)		1	–

* Independent samples *t‐*test.

** Pearson's chi‐square test.

*** Fisher's exact test.

Abbreviations: 95% CI, 95% confidence interval; CSMBS, Civil Servant Medical Benefit Scheme; MSM, men who have sex with men; OR, odds ratio; SD, standard deviation; SSS, Social Security Scheme; TGW, transgender women; UCS, Universal Coverage Scheme.

Table 4 shows the clinical and service outcomes of clients who received in‐person follow‐up and telehealth follow‐up. For clinical outcomes, 12.8% and 3.3% of clients receiving in‐person and telehealth follow‐up, respectively, experienced AEs. Rash was the most common AE; all were grades 1 and 2. Two clients experienced grade 3 dizziness and were managed in‐person. The rates of successful referral to long‐term ART maintenance facility and retention at months 3 and 6 were similar for both groups.

**Table 4:** Comparison of clinical and service outcomes of clients who received in‐person and telehealth follow‐up

	In‐person follow‐up (*N* = 281)	Telehealth follow‐up (*N* = 153)	*p*‐value
AEs	36/281 (12.8%)	5/153 (3.3%)	0.589*
Rash	34/36 (94.4%)	5/5 (100%)	
Dizziness	2/36 (5.6%)	0/5 (0%)	
Referral to long‐term ART maintenance facility among those with 2.5 months of follow‐up time			0.535**
Successful	258/281 (91.8%)	143/153 (93.5%)	
Not successful	23/281 (8.2%)	10/153 (6.5%)	
Retention at month 3 among those reached month 3			0.963*
In care	274/281 (97.5%)	150/153 (98.0%)	
LTFU	5/281 (1.8%)	2/153 (1.3%)	
Discontinued ART	2/281 (0.7%)	1/153 (0.7%)	
Retention at month 6 among those reached month 6			0.148*
In care	192/204 (94.1%)	124/126 (98.4%)	
LTFU	9/204 (4.4%)	1/126 (0.8%)	
Discontinued ART	3/204 (1.5%)	1/126 (0.8%)	

* Fisher's exact test.

** Pearson's chi‐square test.

Abbreviations: AE, adverse event; ART, antiretroviral therapy; LTFU, loss to follow‐up.

Six clients (two heterosexual females, two MSM and two TGW) who completed telehealth follow‐up and nine providers (two physicians, three navigators, one counsellor, one pharmacist, one nurse and one administrative officer) were interviewed. Thematic analysis yielded six themes on the experiences of and feedback on receiving and providing telehealth follow‐up: service access and inequity, cost and time‐saving, confidentiality and stigma, COVID‐19 preventive measures, DSD and service management through teamwork (Table 5).

**Table 5:** Experiences of and feedback on receiving and providing telehealth follow‐up

Themes	Sub‐themes	Quotes
Service access and inequity	Transport challenge	“[The telehealth follow‐up] was great. I lived in other province and it was inconvenient for me [to travel] to pick up my medication in Bangkok”. – MSM client 1
	Time limitation	“Follow‐up via video call was easy and convenient. It's suitable for people who have to travel long‐distance or have limited free time. They can access the follow‐up service without taking a leave from work”. – MSM client 2
	Inexperienced technology users	“Sometimes the clients gave the wrong [LINE] ID or they didn't know their own ID because their children or grandchildren set it up for them….So when we asked for their ID, they could not give it to us, and some of these clients decided they would just come to the clinic [for the in‐person follow‐up] instead”. – Peer navigator 1
	No access to tools to conduct telehealth	“We [offered] telehealth follow‐up to all clients but some clients could not choose this option because they didn't have a smartphone or internet, or they didn't have a suitable space for conducting video call, so these clients would just come to the clinic [for follow‐up]”. – Physician 1
	Financial burden brought by COVID‐19	“Some clients had [financial] problem because the economic crisis during the COVID‐19 pandemic made them short of money. There were many clients like this but they didn't tell us about their situation, and we kept reminding them [to transfer] the fee [for ART delivery] every day”. – Peer navigator 1
Cost and time‐saving	Reduce transport cost	“[Telehealth follow‐up option] saves the overall cost for HIV treatment, including [the cost to] travel to the healthcare facility”. ‐ Administrative officer
	Reduce time spent in the clinic	“The telehealth integrated same‐day ART initiation service is appropriate for the new normalcy of [service delivery during] the COVID‐19 pandemic. It's very beneficial and convenient for clients … for instance, it reduces the waiting time in the clinic”. – Nurse
	Reduce opportunity cost	“The pro [of telehealth follow‐up visit] is that the clients don't have to take time off work, which meant that it doesn't impact with their bonus payment and doesn't cause problem for those who have just started a new job”. – Peer navigator 1
Confidentiality and stigma	Privacy and confidentiality	“[The telehealth follow‐up] provides a sense of privacy for people living with HIV, especially for those who don't feel comfortable going to see a physician [at the clinic] because they don't want to be around other people or are concern about running into someone they know at the clinic. Therefore, being able to consult with the physician via telehealth can help keep their secret”. – Pharmacist
	Judgement from society	“ART client should have the option to receive the service that is private in order to help reduce problems of social pressure and stigma from some healthcare providers”. – Peer navigator 1
	Data security	“I want to see a development of a [new telehealth] platform that we can use instead of LINE application to increase the security and anonymity [of client data]”. – Physician 2
COVID‐19 preventive measures	Clinic decongestion	“[Telehealth follow‐up] helps reduce the number of clients in the clinic, which is appropriate for the ongoing COVID‐19 pandemic”. – Pharmacist
	Avoiding non‐essential travel	“Telehealth follow‐up stops clients from having to travel [to the clinic] and this helps improve the access to ART and medical services”. – Counselor
Differentiated service delivery	Client‐centred design	“We needed to find a way for clients to get their ART and receive the [medical services] as if they came to the clinic. This led to the [incorporation of] the telehealth follow‐up via video call. We chose the technological tools that are widely available, which are smartphone and LINE application. If the clients could not do telehealth because they didn't have a phone or internet, they could still come to the clinic, given how small the outbreak was in our country”. – Physician 1
	Client preference	“During [the first wave of] COVID‐19 outbreak, there was a recommendation to limit non‐essential travel so many clients chose the telehealth follow‐up option. After [the first wave], more clients chose to come to follow‐up at the clinic. Most clients that lived in Bangkok and most clients that wanted to see a doctor face‐to‐face preferred to [come to the clinic] for a one‐stop service, meaning [see a doctor and] refill their medication in one‐go. There were not many clients that chose the telehealth follow‐up after [the first wave] because many clients preferred to talk to the doctor in‐person over via video call”. – Peer navigator 3
Service management through teamwork	Service orchestration	“When [administrative officer's name] came into help…. she could manage everything because she understood the system and how we all worked. We [navigators] only had to…send a summary of clients in each day to her via email with the e‐receipt and prescription attached…and [administrative officer's name] would work with the finance team … and the pharmacist team, so all the steps are linked … and that made a good working system”. – Peer navigator 1
	Provider network	“Some clients who initiated ART on the same day but did not want to refill ART at their registered hospital [were referred to Public Health Center 28], and some clients that could not receive same‐day ART initiation had to start ART at the Public Health Center 28…. Some of these clients might live in other province and the provincial borders were close [during the COVID‐19 outbreak]. So, I planned with the Public Health Center 28 team … that I would mail the ART to the clients, their staff would follow up with the clients, the doctor would prescribe the medication, and the center would cover all mailing costs”. – Peer navigator 2
	Difficulty scheduling a video call	“I had to mediate [between doctors and clients]. Sometimes the client was ready [for a video call] and I didn't understand why the doctor would not start the call already, or when the doctor was ready but the client wouldn't pick up the call but they just told me via LINE that they were available”. – Peer navigator 1

Abbreviations: ART, antiretroviral therapy; COVID‐19, coronavirus disease 2019; ID, identification; MSM, men who have sex with men.

## 4 DISCUSSION

To our knowledge, our SDART initiation service is the first differentiated ART initiation model that has integrated telehealth, which makes it suitable for the COVID‐19 era. Our findings show that SDART reach was about 90% throughout the pre‐, during and post‐first waves of the COVID‐19 epidemic. The rates of ART initiation, duration of ART initiation and rates of follow‐up completion improved over time with over 90% successful referral to long‐term ART maintenance facility and retention rates. After the integration of the telehealth follow‐up, about 35% of clients received this option with comparable referral and retention success of over 90%.

High SDART service performance throughout the three periods could be attributed to the integration of the telehealth follow‐up option because it allowed clients who otherwise might not be able to attend the in‐person visit to be eligible, accept SDART initiation and stay in care. However, only about 35% received telehealth. This might be due to the small scale of the first wave of the COVID‐19 epidemic in Thailand and the swift response supported by the existing public health infrastructure [12] that allowed the continuation of some in‐person services. Our clients and providers who were interviewed indicated that having telehealth as an additional option to conduct follow‐up increased the service access, saved time and cost, improved confidentiality and reduced stigma. This might lead to an increase in the follow‐up completion rate, which is in line with existing literature that shows a decline in missed visits with few people missing the telehealth visit [33]. Our providers viewed telehealth as highly appropriate for the COVID‐19 period, as telehealth can help minimize the risk of acquiring COVID‐19 through social contact in clinic setting and during travel [34, 35].

Our regression analysis did not identify any characteristics associated with receiving telehealth follow‐up. This might point to the consistent uptake of telehealth across clients of different ages, populations and socio‐economic backgrounds. However, the thematic analysis revealed that a small group of ageing clients and inexperienced technology users had difficulty accessing telehealth follow‐up. While the telehealth follow‐up further increased the health access to some populations, it might further exacerbate health inequity in others that might be overlooked. A study conducted in the United States prior to the pandemic found that PLHIV who were on ART >10 years, had lower education, had lower income, had higher HIV stigma perception and were unfamiliar with technology were less likely to use telehealth [36]. Another recent study raised a concern regarding telehealth for those without access to high‐speed internet and telephones [37]. These technological difficulties were recognized by our providers, and the option for audio‐only call was posed as a backup plan for those who were unable to participate in video calls. This strategy was used in clinics in the United States as well [33, 35]. Nevertheless, these technological barriers must be further addressed, such as by providing telehealth tools and training on how to use them, to ensure that everyone has the opportunities and confidence to use telehealth.

Our study reported similar proportions of clients receiving telehealth follow‐up during and post‐first waves of the COVID‐19 epidemic. This differed from a trend analysis conducted in the United States that found a shift from heavy/moderate use of telehealth in April 2020 to minimal use in September 2020 [38]. Moreover, the linkage to care experience is critical in laying the groundwork for and facilitating engagement in care [39], and the telehealth follow‐up has altered this experience. Several studies raised concerns regarding telehealth on loss of communication and support [34, 36, 37], and negative consequence on retention and virologic suppression [40]. Nonetheless, our results indicated that the telehealth follow‐up was comparable or even superior to the in‐person follow‐up for the short‐term outcomes (i.e. AEs, referral success and retention at months 3 and 6). Therefore, further study is needed to assess the long‐term effects and usefulness of telehealth. Lastly, the feasibility of telehealth was largely due to the client‐centred design and good management, as well as the coordinated and enabling policies from the local public health agencies [19, 41], which were not available prior. Ongoing policies are needed to preserve and sustain this practice after the end of the pandemic [42]. Cost‐effectivenessstudies are also needed to assess the scalability and facilitate advocacy for telehealth interventions for SDART.

As Thailand faced a worsened COVID‐19 epidemic in 2021, less PLHIV were linked to care. This occurred particularly among those who were diagnosed at non‐ART initiation facilities and those who required OI investigation and/or treatment as referral to healthcare facilities with infectious disease care and ART initiation capability became more challenging as the COVID‐19 epidemic control has been prioritized over HIV treatment. Thus, while the telehealth follow‐up option has shown that it allowed the continuation of SDART initiation service in 2020, more efforts are needed to adapt the ART initiation service to severe epidemic situation, such as by incorporating telehealth for ART initiation, to prevent a delay in linkage to care for PLHIV.

This study has several limitations. Although the telehealth follow‐up proved feasible at a SDART initiation hub in Bangkok, this finding might not be readily applicable to other settings because the first wave of the COVID‐19 epidemic in Thailand was relatively well‐contained and TRCAC did not partake in COVID‐19 testing and treatment actions. To translate this knowledge to other settings, the service model must be further tailored to suit specific implementation environments, including the demographic and health system factors, as well as the intensity of the local COVID‐19 epidemic, to ensure implementation success. The literature we found on the integration of telehealth into HIV care services came from urban settings in developed and developing countries, which could possibly be a literature bias. We chose to use a widely available and free communication application that was already installed on the smartphones of most people in Thailand for the telehealth follow‐up in order to optimize the limited resources and rapidly launch the adapted service. Further service improvement should focus on the security of telehealth communication platform and the equity in accessing telehealth technology. In this analysis, we used routine service data to illustrate the real‐world implementation. As a result, some data and variables were missing, especially in the during and post‐first waves, as the continuation of service delivery was prioritized over the introduction and collection of new variables. An important missing variable was the reach of telehealth follow‐up, which would be a useful piece of information in order to understand its demand. While the qualitative assessment revealed mostly positive feedback on the telehealth follow‐up, the sample was conveniently selected and might not represent all clients and providers, particularly from those clients who did not receive telehealth follow‐up. Further implementation research is needed to document the integration process in order to better translate this knowledge to other implementers.

## 5 CONCLUSIONS

Timely service adaptation allowed telehealth integration into the SDART initiation service and offered follow‐up options that suited the COVID‐19 situation. This resulted in high SDART reach and uptake, reduced ART initiation duration and uptake of the telehealth follow‐up option with favourable short‐term outcomes. While its long‐term outcomes must still be assessed, telehealth has safely improved accessibility to SDART initiation services during the first wave of a relatively well‐contained COVID‐19 epidemic in Thailand. Further service implementation should focus on increasing its inclusivity, training for quality improvement and advocacy for sustainability. Adaptation to other settings requires further tailoring to specific implementation environments to ensure success. Further SDART initiation service adaptation is also needed to allow service continuation during a more severe COVID‐19 epidemic that Thailand faced in 2021.

## COMPETING INTERESTS

All authors declare no competing interests related to this work.

## AUTHORS’ CONTRIBUTIONS

SA and SL drafted the original manuscript. SA, SL and RR developed the analysis plan. SA, NP, ST, PS, PP, NP and RR designed and directed the study. NP, ST, CH, JS and CP implemented the service and led data collection. SA, SL, LS, TP, NP and SS analysed the data. SA, SL, NT, ST, PS, CH, JS, CP, LS, TP, NP, SS, MA, SM, PP, NP and RR read and approved the manuscript. SA and SL revised the manuscript according to comments received. SA, SL, NT, ST, PS, CH, JS, CP, LS, TP, NP, SS, MA, SM, PP, NP and RR have read and approved the final manuscript.

## FUNDING

This study was supported by the US Agency for International Development (USAID) and US President's Emergency Plan for AIDS Relief (PEPFAR) through the Linkages Across the Continuum of HIV Services for Key Populations cooperative agreement (AID‐OAA‐A‐14‐0045) managed by FHI 360.

## Supporting information




**Appendix S1**. Interview questions
