# Supplementary material for: Evaluation of the integration of telehealth into the same‐day antiretroviral therapy initiation service in Bangkok, Thailand in response to COVID‐19: a mixed‐method analysis of real‐world data
Source: J Int AIDS Soc. 2021 Oct 28;24(Suppl 6):e25816. doi: 10.1002/jia2.25816 (PMC8554221; doi:10.1002/jia2.25816)
Supplement: Supplementary file 1 — Appendix S1. Interview questions [file JIA2-24-e25816-s001.docx]

**Interview Questions**

For providers:

1. What do you think about the implementation of SDART telehealth service during and after COVID-19 outbreak?
2. What are the challenges in the implementation of SDART telehealth service?
3. How do you think the SDART telehealth service can be improved?

For clients:

1. How do you feel about having follow-up visit and physical examination via video call?
2. What do you think about refilling ART via mail?
3. Do you have any suggestions to the ART initiation service?
